# HD-ZIP Gene Family: Potential Roles in Improving Plant Growth and Regulating Stress-Responsive Mechanisms in Plants

**DOI:** 10.3390/genes12081256

**Published:** 2021-08-17

**Authors:** Rahat Sharif, Ali Raza, Peng Chen, Yuhong Li, Enas M. El-Ballat, Abdur Rauf, Christophe Hano, Mohamed A. El-Esawi

**Affiliations:** 1 Department of Horticulture, College of Horticulture and Plant Protection, Yangzhou University, Yangzhou 225009, China; rahatsharif2016@nwafu.edu.cn; 2 College of Horticulture, Northwest A&F University, Yangling 712100, China; 3 Fujian Provincial Key Laboratory of Crop Molecular and Cell Biology, Oil Crops Research Institute, Center of Legume Crop Genetics and Systems Biology, College of Agriculture, Fujian Agriculture and Forestry University, Fuzhou 350002, China; alirazamughal143@gmail.com; 4 Key Laboratory of Biology and Genetic Improvement of Oil Crops, Oil Crops Research Institute, Chinese Academy of Agriculture Science (CAAS), Wuhan 430062, China; 5 College of Life Science, Northwest A&F University, Yangling 712100, China; pengchen@nwsuaf.edu.cn; 6 Botany Department, Faculty of Science, Tanta University, Tanta 31527, Egypt; enas.elballat@science.tanta.edu.eg; 7 Department of Chemistry, University of Swabi, Anbar 23430, Pakistan; mashaljcs@yahoo.com; 8 Laboratoire de Biologie des Ligneux et des Grandes Cultures (LBLGC), INRAE USC1328, Université d’Orléans, 28000 Chartres, France; hano@univ-orleans.fr

**Keywords:** abiotic stress, biotic stress, crop improvement, HD-ZIP, plant development

## Abstract

Exploring the molecular foundation of the gene-regulatory systems underlying agronomic parameters or/and plant responses to both abiotic and biotic stresses is crucial for crop improvement. Thus, transcription factors, which alone or in combination directly regulated the targeted gene expression levels, are appropriate players for enlightening agronomic parameters through genetic engineering. In this regard, homeodomain leucine zipper (HD-ZIP) genes family concerned with enlightening plant growth and tolerance to environmental stresses are considered key players for crop improvement. This gene family containing HD and LZ domain belongs to the homeobox superfamily. It is further classified into four subfamilies, namely HD-ZIP I, HD-ZIP II, HD-ZIP III, and HD-ZIP IV. The first HD domain-containing gene was discovered in maize cells almost three decades ago. Since then, with advanced technologies, these genes were functionally characterized for their distinct roles in overall plant growth and development under adverse environmental conditions. This review summarized the different functions of HD-ZIP genes in plant growth and physiological-related activities from germination to fruit development. Additionally, the HD-ZIP genes also respond to various abiotic and biotic environmental stimuli by regulating defense response of plants. This review, therefore, highlighted the various significant aspects of this important gene family based on the recent findings. The practical application of HD-ZIP biomolecules in developing bioengineered plants will not only mitigate the negative effects of environmental stresses but also increase the overall production of crop plants.

## 1. Introduction

The genes containing the homeobox domain were discovered for the first time in *Drosophila*. This was due to the homeotic mutation, which transformed one part into another part in the *Drosophila* body [1]. Homeobox domain genes are mainly involved in controlling the growth and developmental processes such as transition through phases in an organism by encoding a certain transcription factor [2]. The additional presence of homeodomain (HD), which comprises 60 amino acid sequences and later makes a three-helix tertiary structure, supports the promoter regions to interact with specific target genes [3]. In plants, the first HD-containing gene was reported in maize (*Zea mays*), where a *Knotted1* gene was observed to control the leaf differentiation mechanism. Due to this phenotypic characteristic, the name *Knotted1* was given to this gene and perhaps the first HD family gene from plant genomes [4]. Following that, a series of discoveries reported a large set of genes-possessing HD domain and different other additional domains in a single copy of a gene [5]. These different homeobox gene families exhibit structure and functional similarities [2]. The functional importance of HD-ZIP genes has been documented in a wide range of plant species. For instance, HD-ZIP genes are involved in regulating plant architecture, organogenesis, and reproductive processes [6,7,8]. The aided importance of HD-ZIP genes in curbing environmental stresses is also well highlighted. For instance, most of the HD-ZIP genes in transgenic research showed pronounced effects against drought and salinity [9,10]. Apart from that, these genes respond to various other adverse conditions, including heat, heavy metals, and biotic stresses [11,12]. Therefore, the present review documented several aspects of the homeodomain leucine zipper (HD-ZIP) gene family, such as structural characteristics, interaction with other gene families, and potential in regulating plant growth, development, and responses to environmental cues. 

## 2. Structural Characteristics of HD-ZIP Gene Family

**Figure 1:**
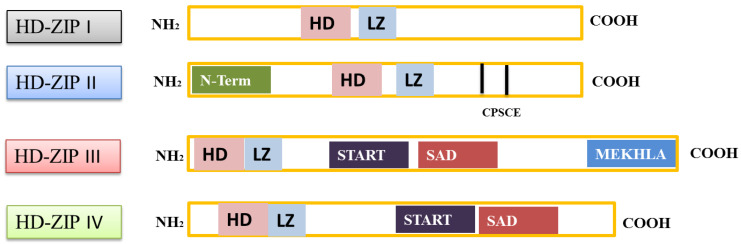
Schematic representation of HD-ZIP genes and their structural distribution split them into four classes (HD-ZIP I, HD-ZIP II, HD-ZIP III, and HD-ZIP IV). HD, LZ, START, SAD, and MEKHLA can be seen in the decoded form in the text.

The HD-ZIP gene family is composed of two functional domains, i.e., HD and leucine zipper (LZ). Based on their sequence conservation and functional properties, HD-ZIP is further divided into four subfamilies (HD-Zip I, HD-Zip II, HD-Zip III, and HD-Zip IV) [13,14]. The subfamily I and II genes encode a small transcription factor (TF) with a similar structure. Both the subfamily I and II consist of a highly conserved HD domain and a contrasting less conserved LZ domain [15,16]. Both class I and class II shared structure similarities; however, some elements are still varied, which differentiate between them. Such as, the HD region of class II contains two introns and three exons and encodes alpha-helixes 2 and 3, whereas, genes in class I comprised one intron at the LZ domain region or alpha-helix 1 [15]. Moreover, an additional Cys, Pro, Ser, Cys, and Glu (CPSCE) motif on the C-terminal differentiates class I from II (Figure 1). The extra motif facilitates the formation of multimeric proteins responsible for the Cys-Cys inter-molecular bond [17]. Further, the class I and II genes showed differences for their specific target sites. For example, the pseudopalindromic sequences CAATNATTG have different central nucleotides A/T and C/G in class I and II genes, respectively. According to an earlier study [18], this class-based target specificity is caused by various amino acids. The amino acids at the alpha-helix 3 (ranging between 46 and 56 nucleotides) are different for class I (ala and trip) and II (Glu and Thr). The changes in these amino acids coupled with Arg55 play a pivotal role during their interaction with DNA molecules [18]. The genes from class I and class II both interact with DNA only in the form of dimers. The strength of the interaction between HD-ZIP proteins and DNA molecules largely depends on the loop region between the first and the second α-helixes and the structure of the N-terminal [19,20].

Likewise, class III and IV genes comprised an additional steroidogenic acute regulatory protein-related lipid transfer (START) domain and a conserved SAD (START-associated domain along with HD and LZ domains). The class III family genes also contain an additional highly conserved methionine-glutamic-lysine-histidine-leucine-alanine (MEKHLA) domain. The MEKHLA domain is unique to class III subfamily genes in plants HD-ZIP gene family [2,21]. The class III MEKHLA domain shares a high similarity with the PAS domain. However, studies are limited over the potential role of the MEKHLA domain in plants [22] besides their involvement in embryo patterning and transportation of auxin [23]. The START domain (~200 amino acid residues) is involved in lipid and sterol transport in animals; however, no study reported their interaction with DNA molecules [24]. On the other hand, no clear evidence of the function of START domain in the plant genome was found. However, the protein-containing START domain could be regulated in plants by lipid/sterol-associated proteins (Figure 1). This regulation could be the outcome of the direct interaction of START domain-containing proteins with lipid/sterol proteins or by third mediator protein [25]. A study supported this notion by reporting that an HD-ZIP class IV gene regulates the phospholipid signaling in arabidopsis roots [26]. Another report concluded that the START domain is essential for the proper functioning of HD-ZIP genes in the cotton plant [27]. The research body is limited regarding class III and IV genes interaction with DNA molecules due to their polymorphic nature. The common distinctive feature of these genes is due to the presence of TAAA sequence in their target sites [2].

## 3. Role of HD-ZIP Genes Family in Plant Growth and Regulation

Numerous HD-ZIP I genes that have evolutionary resemblance generally show the same expression pattern in various plant tissues. For instance, *ATHB1* plays a crucial role in the developmental processes of tobacco (*Nicotiana tabacum*) leaf cells [21]. The transgenic plant overexpressing *ATHB23* or *ATHB3*, *ATHB13*, and *ATHB20* fine-tuned the cotyledon and leaf development processes significantly [15,22]. The ectopic expression of the tomato (*Solanum lycopersicum*) *LeHB-1* gene disrupts the normal flowering process in the transgenic plant [23]. The study also reported that the transgenic plants also resulted in multiple flower production, an abnormal transformation of sepals into carpel and regulates the floral morphogenesis, and triggered the fruit ripening process [23]. Similarly, the grape (*Vitis*) *VvHB58* controls the fruit size, reduced the number of seeds, and hindered the pericarp expansion in the tomato fruit by modulating the multiple-hormones pathway [24]. 

Additionally, this HD-ZIP I TF regulates the growth and development of plants under various adverse conditions. For example, the *HDZI-4* promoter drives DREB/CBF expression under severe drought conditions, which mitigates the negative effects of drought stress and restricts the declination in yield and other growth attributes in wheat and barley [25]. Recently, Ma et al. [26] addressed the crucial role of *ATHB13* in floral induction. Flower induction at an appropriate time is crucial for seed setting, survival, and germination [27,28]. The citrus *PtHB13* is homologous to *Arabidopsis ATHB13*. The ectopic expression of *PtHB13* in *Arabidopsis* inhibited the floral induction process and could regulate the flowering-related genes [26]. Majority of the reports available on HD-ZIP I TFs suggested that they are mostly induced under abiotic stresses and thus crucial for maintaining plant growth under unfavorable environments.

There are nine genes in the *Arabidopsis* HD-ZIP II subfamily. The main role of this class in plant development is their shade-avoiding mechanism during the photosynthetic process [29,30,31]. For example, one member of class II subfamily *ATHB2,* when overexpressed in *Arabidopsis,* unfolded its role in plant development under illumination conditions [32]. On the other hand, microarray analysis revealed that *HAT2,* a member of the class II HD-ZIP gene family, was significantly influenced by the auxin during the seedlings stage [33]. To confirm that, *Arabidopsis* plants overexpressing the *HAT2* gene produced epinastic cotyledons, long hypocotyls, long petioles, and small leaves. All these traits resembled to the mutants, generating auxin in high quantity [33,34]. Fruit ripening is an important qualitative factor that defines the fate market value of postharvest produces. Ethylene is generally considered a potent regulator of the fruit ripening process. In this regard, the overexpression of *PpHB.G7*, a class II HD-ZIP family gene in peach (*Prunus persica*), mediates the ripening process by altering the expression and production of ethylene biosynthesis genes and ethylene, respectively [35]. In a recent study, the rice (*Oryza sativa*) *sgd2* gene was found responsible for small grain size and dwarf plant phenotype. The study further showed that the *sgd2* gene is a transcriptional suppresser of GA biosynthetic genes, particularly suppressing the generation of endogenous GA_1_ [36]. The majority of the class II HD-ZIP genes that are differentially expressed in various plant tissues confer their importance in regulating plant developmental activities. 

*Arabidopsis* genome contains five members of the class III HD-ZIP gene family. Numerous mutants of these genes have been reported previously. Most of the class III genes are responsible for sustaining the normal organ polarity and shoot apical meristem (SAM) [37]. Single loss of HD-ZIP III protein function does not display any obvious phenotypic changes. However, a double or triple mutant of class III genes such as *phb-6/phv-5/rev-9* lacked SAM along with single abaxialized cotyledon, suggesting their overlapping nature [38]. Additionally, overexpression of *Arabidopsis ATHB8* hastened the xylem formation because of the ectopic production of procambial cells [39]. In contrast, loss of function of *ATHB8* failed to show any physiological and morphological changes [39]. The *ATHB15* gained the *icu4-1* function allele, resulting in an abnormal arrangement of root meristem and more number of lateral roots production than the wild type (WT) [40]. Taken together, the aforementioned statements elucidated the crucial role of class III genes in root formation and vascular development. Another study [41] supported the notion by reporting the role of class III gene in nodule formation, root development, and vascular activities regulation. The results highlighted that *GmHD-ZIP III 2* demonstrated strong interaction with *GmZPR3d,* ensuing in the ectopic formation of secondary root xylem and also a dominant expression of soybean (*Glycine max*) vessel-specific genes [41].

The class IV HD-ZIP gene family has been previously characterized in various plants such as *Arabidopsis,* maize, and rice. These genes generally show a dominant expression trend in the outer layer of SAM and the epidermal cells [42,43]. Additionally, these genes are mainly involved in the developmental processes of stomata, trichome and epidermis, cuticle, and root hairs [2]. In line with that, two functionally redundant class IV genes, *ARABIDOPSIS THALIANA MERISTEM LAYER1* (*ATML1*) and *PROTODERMAL FACTOR2* (*PDF2*) in *Arabidopsis,* were reported for their crucial role in regulating the epidermis and embryo development and also in the patterning of floral identity [44,45]. The *TRICHOMELESS1* (GL2) gene in *Arabidopsis,* a member of the class IV gene family, has been recognized for fine-tuning the trichome and root hair development [46]. Anthocyanins are potent regulators of leaf pigments and mainly responsible for protecting chloroplast against deleterious environmental effects [47]. The *Arabidopsis ANTHOCYANINLESS2* (*AtANL2*) controls the deposition of anthocyanins, root growth and ectopic root hairs development, and also epidermal cells proliferation [48,49]. Improved root growth is significant in providing support to the plant in water-scarce conditions. The class IV gene *ATHDG11* led to the overall improvement root system in the overexpressed *Arabidopsis* transgenic plants [50,51]. Apart from *Arabidopsis,* the function of class IV HD-ZIP genes have been in other economically important crops such as rice and maize. The maize *ZmOCL1* and *ZmOCL4* have been reported to regulate cuticle deposition, kernel development, and trichome formation [42,52]. In rice, the *Roc4* gene, a member of the class IV HD-ZIP gene family, manipulates flowering time by regulating the expression of *Ghd7* gene. The results revealed that the overexpressed *Roc4* rice transgenic plants showed repressed expression of *Ghd7* under long days and thus hastened the flower induction processes [53]. Altogether, the aforementioned evidence highlighted that the class IV HD-ZIP gene family has an imposing role in plant growth and developmental activities. 

## 4. The Crucial Role of HD-ZIP Gene Family in Regulating Abiotic Stress

### 4.1. Role of HD-ZIP I Subfamily in Abiotic Stress Control 

Plants adopt various mechanisms to cope with numerous abiotic stresses [54,55]. The HD-ZIP class I genes are generally known for assisting with abiotic stress responses and tolerance, particularly drought, salinity, and cold stress. Thus, in the subsequent sections, we have explained the vital role of HD-ZIP genes-regulating stress-responsive mechanisms under numerous abiotic and biotic cues. Apart from the textual explanation, a large amount of literature has been tabulated and presented in Table 1. 

#### 4.1.1. Drought Stress

Drought is a major stress suffered by plants. It impairs plant physiological and biochemical functions and is considered a major threat to food security in the current time [56,57]. The *AtHB7* and *AtHB12*, two paralogous genes, induced significantly under ABA and water stress conditions by regulating stomata closure [58,59]. The *Oshox4* interacted with DELLA-like genes and further regulated the gibberellic acid (GA)-signaling pathway that confers drought stress tolerance in rice [60]. Additionally, the rice *Oshox22* showed dominant transcriptional activities under the prolonged drought stress [16]. The sunflower (*Helianthus annuus*) *Hah-4* gene was overexpressed in the maize plants to elucidate its role in mitigating the drought stress. The study revealed the crucial role of *Hah-4* gene in increasing the resistance of maize plants against drought stress without hindering the agronomic traits and colonization of root Arbuscular mycorrhizal fungi activity [61]. The accumulation of ABA in the leaf is significant and plays a key role in maintaining normal plant growth under drought stress [62]. The *Nicotiana attenuata* class I HD-ZIP gene *NaHD20,* when overexpressed, facilitates the ABA accumulation in leaf under water-scarce conditions, which also triggered the expression level of dehydration responsive genes such as *NaOSM* [62]. On the contrary, the *NaHDZ20* gene-silenced plants displayed increased susceptibility to drought stress. The reduction in the NaHDZ20-silenced plants’ drought tolerance could be attributed to the suppressed expression level of dehydration responsive genes [62]. The wheat (*Triticum aestivum*) gene *TaHDZ5-6A* was overexpressed in *Arabidopsis.* The transgenic *Arabidopsis* plants generated high proline contents, better water holding capacity, and a good survival rate under drought stress than the wild-type plants [9]. This growing evidence confirmed the role of HD-ZIP I subfamily genes in maintaining plant growth under water deficit conditions.

#### 4.1.2. Salinity Stress

Around 40 million hectares of world irrigated arable land are affected by salinity, which causes massive economic losses to the countries with the worst sodic soil [63]. Salt stress or salinity affects the plants when the soil NaCl content is more than the required amount [64,65]. The HD-ZIP I subfamily genes have been reported for their mitigatory role against salt stress in plants [59]. For example, the *AtHB1* induced strongly under salinity stress in *Arabidopsis* [15]. Similarly, the rice *OsHOX22* gene restored resistance significantly against prolonged NaCl stress by mediating the ABA signaling machinery [66]. Two genes from *Craterostigma plantagineum* (*CpHB6* and *CpHB7*) simultaneously curb the drought and salinity stress by showing an induced expression trend in roots and leaves [67]. The *GhHB1* gene has been functionally characterized in cotton (*Gossypium hirsutum*) plants. A remarkable increase in the expression activity of *GhHB1* gene was observed under 1% NaCl stress [68]. The results further revealed that the transgenic cotton plants showed enhanced resistance to salinity stress by modulating the root developmental processes [68]. The maize *ZmHDZ10* was overexpressed in rice. The transgenic rice plants hastened their resistance against salinity by triggering the production of proline while alleviated the malondialdehyde (MDA) activities in comparison to that of wild type [69]. In a recent study, the *JcHDZ07* gene was isolated from physic nut (*Jatropha curcas*) and overexpressed in the *Arabidopsis.* The transgenic *Arabidopsis* plants showed increased sensitivity to salinity stress by exhibiting higher electrolyte leakage activities, lower proline content, and hindered antioxidant activities [70]. Taken together, these results suggested the important regulatory role of the HD-ZIP I subfamily in plants against salinity stress. 

#### 4.1.3. Low-Temperature Stress

**Figure 2:**
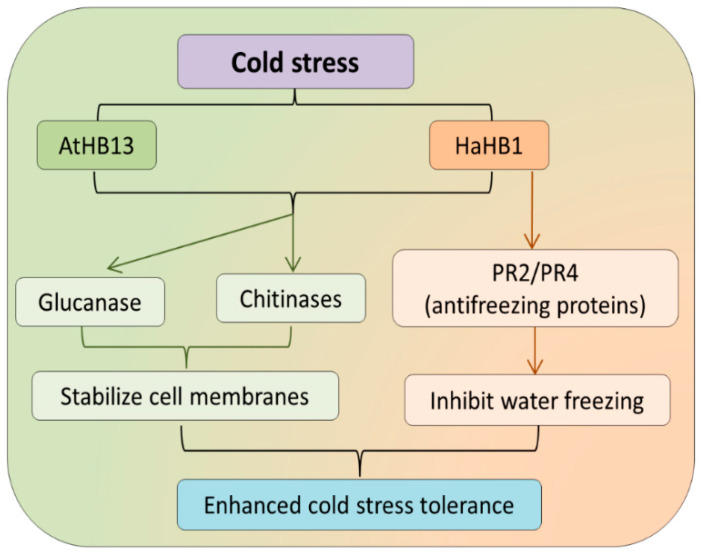
Role of HD-ZIP I subfamily in regulating low-temperature stress. The cold stress induces *AtHB13* and *HaHB1* gene, which further activates the transcription of chitinases, glucanase, and PR2 genes. These genes help stabilize the water transport and inhibit it from freezing inside cell membrane.

Low-temperature stress alters the photosynthetic, ions transport, and metabolic activities by directly targeting the cell fluidity [71,72,73]. Plants use different mechanisms and signaling pathways to deal with low-temperature stress. In this regard, HD-ZIP I subfamily genes have been characterized in various plants and yielded significant results. For example, the wheat *TaHDZipI-2* was overexpressed in barley resulted in the acclimatization of barley plants to cold conditions. The overexpressed transgenic plants also exhibited better flowering under low temperatures than the wild type [74]. Similarly, the *TaHDZipI-5* showed upregulated expression trends in flowers and grains. Further, under low temperature, *TaHDZipI-5* indicated its role in cold tolerance during the reproductive stage [75]. To confirm that, transgenic wheat plants overexpressing the *TaHDZipI-5* restore the normal flowering activities under cold stress; however, compromised agronomic and yield-related traits were observed [75]. Overexpression of the *AtHB13* gene confers cold stress tolerance by maintaining cellular stability in *Arabidopsis* plants [59]. The expression level of several glucanase, anti-freezing proteins (AFP), pathogenesis-related proteins, glucanase, and chitinase enhanced significantly in the *HaHB1* sunflower and soybean transgenic plants showed improved resistance to cold stress [76]. Therefore, it is confirmed that the HD-ZIP I subfamily genes facilitate the resistance mechanism against cold stress by triggering the expression of the cell membrane-related proteins and AFP (Figure 2).

#### 4.1.4. Heavy Metal Stress

The increasing soil pollution with heavy metals, such as cadmium, chromium, iron, lead, nickel, selenium, etc., causes toxic reactions that hamper the physiological and morphological activities of plants [77,78,79]. Recent studies have reported the involvement of HD-ZIP I genes in regulating heavy metals stress. In *Citrus sinensis*, for example, the cDNA-AFLP methodology revealed that two genes from HD-ZIP I subfamily (*TDF #170-1* and *170-1k*) enhanced significantly under manganese (Mn) toxicity, suggesting their possible role in Mn stress tolerance [80]. Based on this, it could be of high interest to elucidate the role of these genes under various important toxic heavy metals.

#### 4.1.5. Heat Stress

The rise in global temperature is becoming increasingly challenging to crop scientists as heat stress causes early maturity of the plants and subsequent manifold reduction in overall yield [55,81,82]. Expression-based analysis in cucumber (*Cucumis sativus*) suggested that two members (*CsHDZ02* (*Csa1G045550*) and *CsHDZ33* (*Csa6G499720*)) of HD-ZIP subfamily I showed induced expression pattern under heat stress [83]. The sunflower *HaHB4* gene has been functionally characterized in soybean plants under field conditions [11]. The transgenic soybean plants overexpressing *HaHB4* genes exhibited better tolerance capacity to heat stress by triggering the transcriptional activity of heat shock proteins (*AT-HSC70-1, AT-HSFB2A,* and *Hsp81.4*) [11]. On the other hand, *HaHB4* transgenic plants recorded better yield by reducing heat stress damage during seed setting in soybean pods [11]. The perennial ryegrass (*Lolium perenne*) is generally regarded as heat-sensitive because of its temperate growth nature [84]. Perennial ryegrass is mostly grown for turf or forage purposes; however, increasing temperature due to global warming hampered its production manifold [84]. The HD-ZIP I subfamily gene *LpHOX21* possessed upregulated expression in the heat-tolerant cultivar of perennial ryegrass, which suggested its possible involvement in enhancing resistance to heat stress [84]. Although the HD-ZIP subfamily I genes are well characterized under other abiotic stresses, still, relatively less research is available regarding their role in mitigating heat stress. 

#### 4.1.6. Flooding Stress

Flooding stress refers to the plant’s submergence, which creates an anaerobic condition in the surroundings and affects plant productivity [85,86]. The HD-ZIP I subfamily gene *HaHB11* was overexpressed in the *Arabidopsis* and exposed to flooding stress [87]. The transgenic *Arabidopsis* plants carrying gain of function *HaHB11* gene induced the tolerance to flood stress and increased the biomass and yielded more seeds than control [87]. Flooding is becoming a serious threat due to climate change, and therefore the HD-ZIP TFs could be utilized to generate flooding resistance cultivars.

#### 4.1.7. Nutrient Stress

Excess or deficiency of plant nutrients in the soil is generally regarded as nutrient stress. The transition heavy metals such as manganese, zinc, copper, and iron are essential micronutrients for regulating the plant’s growth and developmental activities [88,89]. Iron in a relatively small amount is considered an important nutrient and involves key regulatory processes (chlorophyll biosynthesis and photosynthesis) of plant development [90]. Higher plants solubilize the ferric iron in the rhizosphere region, facilitating the uptake of iron efficiently [91]. The HD-ZIP I subfamily member gene *AtHB1* was previously reported for its involvement in iron homeostasis [92]. The lack of function *athb1* gene showed strong tolerance to iron deficiency by upregulating the expression of *Iron-Regulated Transporter1* (*IRT1*) and exhibited higher chlorophyll contents than the control [92]. In contrast to that, the overexpression of *AtHB1* genes suppressed the transcription activity of *IRT1* genes, which hampered the plant’s iron regulation, indicating a crucial role of the *AtHB1* gene in iron homeostasis [92]. This suggested the importance of HD-ZIP I subfamily genes in maintaining the uptake and translocation of iron and other essential nutrients and could be used as a genetic tool to improve crop productivity and nutrient efficiency.

### 4.2. Role of HD-ZIP II Subfamily in Abiotic Stress Control

#### 4.2.1. Drought Stress

The HD-ZIP II subfamily is renowned for providing resistance against important abiotic stresses such as drought, cold, and salinity stress. The *SiHDZ13* and *SiHDZ42* showed upregulated transcriptional activity under prolonged drought stress in the sesame (*Sesamum indicum*) plant [93]. In another study, the expression of wheat *Tahdz4-A* strongly increased under drought stress, conferring its responsive nature to this important abiotic stress [94]. Similarly, in *Arabidopsis,* increased mRNA level of *HAT2* and *HAT22* genes was observed under water deficit conditions [95]. Eucalyptus is an industrial plant and generally used for paper and timber production [96]. However, its production has been affected and reduced significantly by water scarcity [97]. The gain of function *EcHB1* gene significantly boosted the photosynthetic capacity, which increased the number of chloroplast unit per leaf area under drought stress in transgenic eucalyptus plants [98]. Numerous expression studies suggested the importance of HD-ZIP II subfamily genes in regulating drought stress. However, the smaller number of functional studies encourages future research over HD-ZIP II subfamily genes in various important plants.

#### 4.2.2. Light Stress

The vast number of HD-ZIP II subfamily genes across different plant species has been reported to respond to light stress, and shade avoidance in particular [99]. The *AtHB2/HAT4* is strongly induced under the dark condition in the etiolated seedlings [100]. To confirm their function, transgenic lines overexpressing *AtHB2/HAT4* produced longer hypocotyls [101]. This indicated that *AtHB2/HAT4* is responsible for controlling the growth of seedlings in fluctuating light conditions. Additionally, the *AtHB2* (protein) directly interacts with PIF proteins because the expression was completely lost in *pif4, pif5,* and *pifq* [102,103,104]. The ectopic overexpression of various HD-ZIP II subfamily members could phenocopies the positive shade avoidance effects over other organs such as flowers [31]. Light stress is controlled by multiple pathways and, therefore, further studies are required to unfold the potent role of HD-ZIP II subfamily genes in plants.

#### 4.2.3. Salinity Stress

The HD-ZIP II subfamily has been examined extensively in different plants under salinity stress. However, functional characterization of these genes under NaCl stress is far little compared with subfamily I genes. The tea (*Camellia sinensis*) *CsHDZ15* and *CsHDZ16* increased significantly throughout the stress period, suggesting that they are involved in responding to salinity stress [105]. The *StHOX17, StHOX20,* and *StHOX27* genes possessed dominant expression under the saline condition in potato (*Solanum tuberosum*) plants [106]. Additionally, the *Capsicum annum* (*CaHB1*) showed an enhanced expression trend under various stresses, including salt. To verify its role, the *CaHB1* gene was overexpressed in tomato plants. The transgenic tomato plants displayed improved resistance against NaCl stress. Moreover, the transgenic tomato plants developed better agronomic traits than the wild type [10]. These results imply the beneficial roles of HD-ZIP II subfamily genes in mitigating the salinity stress and could be useful in future crop-breeding programs. 

### 4.3. Role of HD-ZIP III Subfamily in Abiotic Stress Control

#### 4.3.1. Drought Stress

The members of HD-ZIP III subfamily are mainly involved in the leaf-rolling mechanism of plants. Leaf rolling is an important factor that provides assistance to plants under water deficit conditions. The HD-ZIP III subfamily genes are the major target genes of *miRNA165/166*. In line with that, rice miRNA166 loss-of-function mutant (STTM166) developed rolled leaf phenotype because of damaged sclenrenchymatous cells along with abnormal bulliform cells [107]. The molecular dissection of the STTM166 mutant revealed that the *OsHB4* gene is targeting the *miRNA166*, a member of the class III HD-ZIP gene family [107]. The *miRNA166* STTM166 mutant lines showed enhanced resistance to drought stress. To validate that, *OsHB4* overexpressed transgenic rice plants influenced the expression of polysaccharide synthesis genes, which facilitates the cell wall and vascular developmental activities and also imposed the rolled leaf phenotype conferred tolerance to drought stress [107]. In another study, the *Arabidopsis miRNA160/166* double mutant displayed enhanced resistance under water-scarce conditions by influencing the expression of auxin-related genes and exhibiting rolled leaf phenotype [108]. Based on the above-mentioned shortcomings, it can be concluded that the HD-ZIP III subfamily genes also possessed drought stress-responsive factors and, therefore, can be considered to characterize in different plants apart from model species. 

#### 4.3.2. Salinity Stress

Studies based on expression analysis suggested that HD-ZIP III subfamily genes are responsive to salinity stress. For example, the wheat HD-ZIP III genes *Tahdz1* and *Tahdz23* both induced under NaCl stress [94]. The *MtHDZ5, MtHDZ13,* and *MtHDZ22* showed differential expression under 180 mM and 200 mM NaCl stress in 2-week old seedlings of *Medicago truncatula* [109]. However, research is required to elucidate the functional role of these genes under saline conditions in numerous plant species. 

#### 4.3.3. Heavy Metal Stress

Heavy metal toxicity is consistently hampering plant productivity due to the increasing environmental pollution. They generally compromised plants’ physiological and molecular pathways and caused irreparable damage [78,79]. Among them, cadmium (Cd) is a highly toxic metal that has been reported for causing yield losses in various plant species [78,110,111,112]. The application of Cd induced the expression of rice *OsHB4,* whereas the miRNA166 was deduced under Cd treatment [12]. This suggested the possible involvement of this gene in regulating Cd stress in rice. To confirm this, an overexpression assay was performed for miRNA166. The overexpression of miRNA significantly reduced the transcriptional activity of *OsHB4* in root and leaf tissue. On the other hand, the miRNA166 was strongly induced in both the root and leaf and hindered the Cd translocation from root to stem [12]. Additionally, the accumulation of Cd in the rice grain was also arrested in the miRNA166 overexpressed transgenic lines. On the contrary bases, the overexpression of *OsHB4* made the root and leaf more sensitive to Cd toxicity, whereas RNAi silencing of *OsHB4* made the transgenic plants tolerant to Cd stress [12]. These evidences clearly suggest that the induced expression of *OsHB4* increased the rice plant’s sensitivity to Cd stress. Furthermore, the majority of the HD-ZIP III subfamily genes showed pronounced expression during root development-related activities [113,114]. Therefore, this could be vital in providing stress response to heavy metals. 

### 4.4. Role of HD-ZIP IV Subfamily in Abiotic Stress Control

#### 4.4.1. Drought Stress

The HD-ZIP IV subfamily genes have been recently characterized in many plants to induce drought stress tolerance in plants. The gain of function *OsHDG11* gene (a member of HD-ZIP IV) enhanced the overall yield and drought stress tolerance mechanism in rice plants. The transgenic rice plants overexpressing *OsHDG11* significantly influenced the root system, improved water holding capacity, triggered the proline content, and enhanced endogenous ABA production [51]. Similar results were found when the *AtEDT1/HDG11* gene was overexpressed in Chinese kale; however, altered endogenous ABA imposed stomatal closure [115]. Lignification in the plant is associated with an array of abiotic stress tolerance. The *Oryza sativa transcription factor I-like* (*OsTFIL*) gene has been reported for its beneficial role in providing tolerance against drought stress in rice. The transgenic rice plants carrying *OsTFIL* gene showed enhanced lignin accumulation in shoot tissue than the RNAi or WT plants [116]. Additionally, the high transcriptional activity of lignin biosynthesis genes also facilitates stomatal closure under drought stress [116]. This *HDG11* gene was further reported in cotton to induce water use efficiency (WUE) and improved stress tolerance [117]. A recent study investigated the genetic pathway of the *HDG11* gene on how it facilitates the WUE and tolerance of a plant to water stress conditions. The study unfolded that the genetic pathway consists of *EDT1/HDG11, ERECTA*, and *E2Fa* loci. Initially, *ERECTA* become transcriptionally activated by binding with HD element in its promoter region. *ERECTA* then modulates the transcription of cell-cycle pathway genes, which further helps in the transition of mitosis into endocycle. This mechanism positively affected the leaf cell size by triggering the ploidy level, which in turn altered the stomatal density [118]. The reduced density of stomata modulates the WUE system of plants and thus provides resistance against drought stress. Other members of this subfamily also showed a response to drought stress, such as that in *Nicotiana tabacum.* The *NtHD-ZIP IV 4* and *NtHD-ZIP IV 10* displayed a dominant expression trend under prolonged drought stress conditions [119]. 

#### 4.4.2. Salt Stress

Cotton crop, although known as a moderate salt-tolerant crop, is still affected by salinity [63]. Salt stress causes a substantial delay in flowering, which implies less fruiting and decreased cotton ball weight [63,120]. The effect of salt stress is more pronounced during the germination and seedling stage of cotton [63,120]. The HD-ZIP IV gene *AtEDT1/HDG11* restored the cotton plant resistance to salt stress by the induction of proline and soluble sugar contents along with an improved antioxidant enzymes system [117]. Remarkably, the transgenic cotton plants showed no compromised agronomic traits and thus yielded more numbers of cotton balls per plant than the wild type [117]. Exogenous application of jasmonic acid (JA) on plants ameliorates the deleterious effects of many abiotic stresses, including salt stress [121,122]. The *EDT1/HDG11* gene was overexpressed in *Arabidopsis,* which hastened the transcriptional level of numerous JA biosynthetic genes and influenced the formation of lateral root significantly by activating the auxin signaling pathway [123]. The endogenous JA level was also high in the roots of transgenic plants [123]. The above statement suggested that the *EDT1/HDG11* transgenic plants could be resistant to multiple environmental stresses, including salt stress.

#### 4.4.3. Osmotic Stress

Osmotic stress dysfunction affects plants’ normal physiological processes by disturbing the transport of ion and water [124]. The cotton *GaHDG11* gene was overexpressed in the *Arabidopsis* plant. The transgenic *Arabidopsis* plants showed better performance under osmotic stress because of the high generation of osmoprotectants such as proline, enhanced antioxidant activities, and elongated roots [125]. The elongation of primary roots supports the plant by lowering the rate of water loss [125]. Due to these noticeable functional characteristics, more research is required to functionally elucidate the role of HD-ZIP IV subfamily genes under osmotic stress.

## 5. Role of HD-ZIP Gene Family in Regulating Biotic Stress

Climate change made not only abiotic stresses but also biotic stresses more challenging for plant scientists. Often, fluctuations in temperature or water stress directly trigger biotic stressors’ negative response and do irreversible damage to the plants [132,133]. The positive roles of HD-ZIP genes in mitigating abiotic stresses have been discussed above. Besides, the HD-ZIP genes could play a powerful role in amending the deleterious effects of biotic stresses (Table 2). In this context, these myriad biomolecules could be utilized to curb the simultaneous stresses (biotic and abiotic). Below, we discussed the roles of HD-ZIP genes in arming the plants against biotic stresses. 

### 5.1. HD-ZIP I: Role in Coping Biotic Stress

Biotic stresses generally affect the plant morphologically and physiologically, which can be challenging to control at times [134,135,136]. For example, the powdery disease infecting numerous crops worldwide cost millions of dollar to the economy [137]. The HD-ZIP I subfamily member *AtHB13* increased *Arabidopsis* plants’ resistance to powdery mildew fungi by regulating the expression of many stress-specific TFs. In contrast, the silencing of *AtHB13* increased the sensitivity of *Arabidopsis* to powdery mildew disease [138]. These results supported the notion that *AtHB13* might be involved in providing resistance against simultaneous abiotic and biotic stresses [138]. The *HAHB4* expression is strongly induced under the herbivores attack or jasmonic acid (JA) treatment. The induced expression produced green leaf volatiles and trypsin protease inhibitors (TPI). The overexpression of *HAHB4* in *Zea mays* and *Arabidopsis* triggered the transcript level of stress-related genes such as lipoxygenase and TPI. The lipoxygenase and TPI genes in plants provide a protective response to *Spodoptera littoralis* or *Spodoptera frugiperda* larvae [139]. Additionally, the transgenic plants overexpressing *HAHB4* generated a higher amount of JA, JA-isoleucine, and ethylene (ET), which lead us to assume that this gene could enhance the resistance against biotic stress casual agents [139]. The *Verticillium dahlia* is a fungal pathogen that is responsible for vascular wilt disease in a plethora of plant species, including cotton. JA has been previously reported for enhancing the resistance of cotton plants to *Verticillium dahlia* [140]. In line with that, the overexpression of the *GhHB12* gene suppressed the transcriptional activities of JA biosynthesis and responsive genes (*GhJAZ2, GhPR3*). It thus made the cotton plant more susceptible to *Verticillium dahlia* fungus [141]. Minimal research is available on the role of HD-ZIP I subfamily genes in mitigating biotic stresses in comparison to abiotic stresses. However, it could be of great interest to functionally characterize these genes under various biotic stresses.

### 5.2. HD-ZIP II: Role in Coping Biotic Stress

The HD-ZIP II subfamily members are investigated under various biotic stress and showed differential expression patterns in several plant species. The *Phytophthora infestans* (*P. infestans*) is a bacterial pathogen, which causes the late blight disease particularly in potato and tomato, and becomes a major challenge for many crop producers around the world [142,143]. The potato *StHOX28* and *StHOX30* exhibited high expression under the P. *infestans* stress. This suggested their responsive behavior toward biotic stresses [106]. The *Phytophthora capsici* (*P. capsici*) is a multi-host fungus pathogen with more drastic effects on Solanaceae (pepper and tomato) and Cucurbitaceae (cucumber and pumpkin) [144,145]. The overexpression of *Capsicum annuum* HD-ZIP II gene *CaHB1* in tomato increased the thickness of cell wall and cuticle layer, enhanced expression of defense genes (*SlPR1, SlGluA, SlChi3,* and *SlPR23*), and larger cell size than the control plants conferred tolerance to *P. capsici* [10]. Therefore, HD-ZIP II subfamily genes could be considered for potential crop improvement in the future.

### 5.3. HD-ZIP III: Role in Coping Biotic Stress

Expression analysis-based studies revealed that the HD-ZIP III genes of potato *StHOX7, StHOX16, StHOX26,* and *StHOX38* showed upregulated expression trend under *P. infestans* stress [106]. The *Arabidopsis AtHB8* genes induced significantly at 5 and 7 days post-inoculation (dpi) of root-knot nematode (RKN) *Meloidogyne incognita* [146]. The *AtHB8* plays an important role in the root developmental activities and, therefore, could be a potential candidate gene in providing a gateway to RKN to form gall around the root [146]. The *PHB* and *PHV* genes of the *Arabidopsis* class III family are responsible for the upward curled leaf phenotype. Similar characteristics were shown by the plants when treated with *Tomato yellow leaf curl China virus* (TYLCCNV) [147]. The results were confirmed in βC1 (pathogenesis protein) overexpressing transgenic plants, which showed an increase in the mRNA level of *PHB* and *PHV* genes while suppressed the expression of miRNA166 [147]. Therefore, it can be suggested that *PHB* and *PHV* play a crucial role in regulating the response of plants to TYLCCNV. However, no conclusive evidence is available to confirm the role of *PHB, PHV,* and other members of HD-ZIP III genes under TYLCCNV or other biotic stress casual agents. 

### 5.4. HD-ZIP IV: Role in Coping Biotic Stress

**Figure 3:**
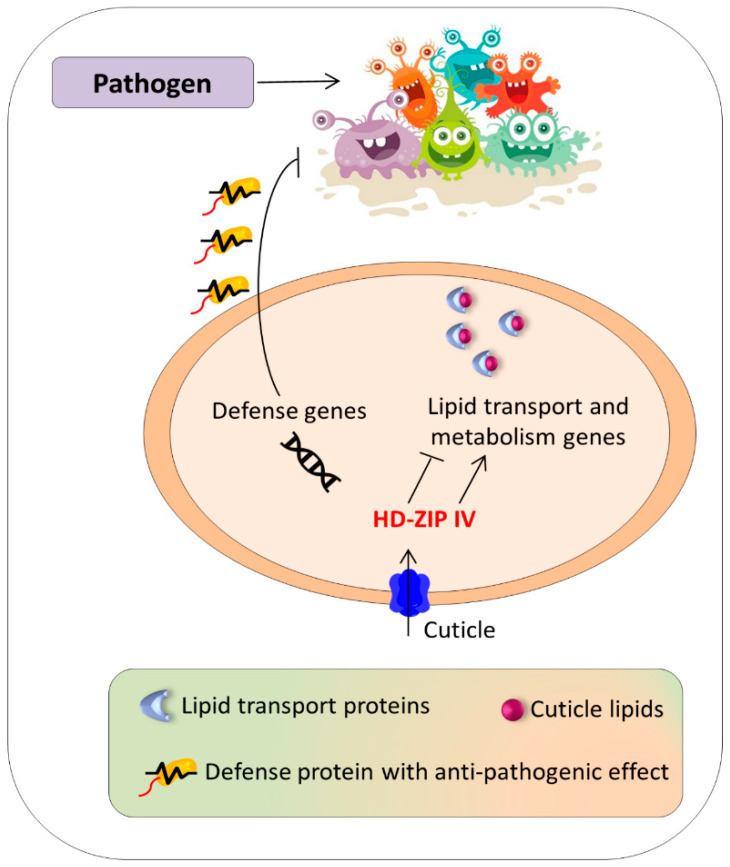
Indirect involvement of HD-ZIP IV subfamily in enhancing the resistance to biotic stress. The HD-ZIP IV genes participate in the activation of cuticle formation and defensins genes. The induction/suppression of lipid transport and metabolism genes largely depends on the HD-ZIP IV genes. The majorities of these genes reside in the epidermis and work synergistically in responding to pathogens.

The cuticle layer in plants provides support against many abiotic stresses. Several reports also highlighted that these cuticle layer films around plant cells serve as the first line of defense against pathogen attack [148,149,150]. The activation of the HD-ZIP IV gene *AaHD8* strongly induced the expression of cuticle development-related genes and significantly affected cuticle formation processes in the Artemisia annua plant [151]. The study also revealed that *AaHD8* interacts with the *AaMIXTA1* gene (regulator of cuticle formation), modulating the *AaHD1* transcription and regulating a network of other cuticle developmental genes [151]. The phenols present inside a trichome generally provide a chemical barrier to the invading pathogen and protect the plant from drastic damage, particularly from chewing pests, such as herbivores [152]. The HD-ZIP IV gene *AaHD8* and *CmGL* were reported for their potent role in trichome formation and development in Artemisia annua and melon plants, respectively [151,153]. The *ZmOCL1* (member of HD-ZIP IV) gene was overexpressed in *Zea mays.* The transgenic maize plants showed induction in the expression of *LIPID TRANSFER PROTEIN TYPE 2* (*nsLTPII*)*, CARBOXYLESTERASE* (*AtCXE-18*)*,* and *PHOSPHATIDYL INOSITOL TRASNPORT PROTEIN* (*SEC14*) [42]. Among them, the *LTPII* is crucial in the transportation of cuticle lipids across the cell wall [154]. These LTP genes were also reported to increase resistance against a plethora of biotic stresses. These proteins belong to the plant defensins family and exhibit remarkable antifungal and antibacterial ability [155,156]. These genes are generally expressed in the outer layer or epidermis [156,157], the same as the HD-ZIP IV subfamily genes. The durum wheat *TdGL7* gene under wounding stress elevated significantly in the grain tissue, similarly to the defensins genes [158]. This provides potential grounds for the biotechnological manipulation of the *TdGL7* gene in wheat to protect the grain from chewing insects or fungi [158]. Therefore, it can be assumed that the HD-ZIP IV subfamily genes could be indirectly involved in regulating biotic stresses (Figure 3). Moreover, the potato *StHOX21* and *StHOX42* increased manifold under *P. infestans* stress [106]. However, no functional study is available to confirm the direct involvement of HD-ZIP IV subfamily genes in increasing tolerance against biotic factors. 

## 6. Conclusions and Future Perspective

The HD-ZIP is an important gene family involved in the diverse roles of plant growth and developmental activities. Apart from their role in plant growth, several studies proved the potential of HD-ZIP genes in enhancing plant tolerance to various abiotic and biotic stresses. For example, the HD-ZIP I subfamily genes are involved in responding to drought and salinity stress in particular, whereas significant results were achieved in transgenic plants against various biotic stresses. The HD-ZIP II subfamily genes protect the plants from the deleterious effects of a shade. A member gene of HD-ZIP II subfamily CaHB1 was also reported for providing resistance against *P. capsici* and salt stress. The HD-ZIP III subfamily genes are characterized mainly under drought stress; meanwhile, another study [12] showed that the silencing of the *OsHB4* gene induced the plant immunity against Cd stress. Additionally, the HD-ZIP IV subfamily genes are mainly expressed in the outer cell membrane and provide the first line of defense against different environmental stresses. 

Further investigations are still required to characterize the function of these important TFs under numerous abiotic (heat, heavy metals, flooding, and nutrient imbalance) and biotic (powdery mildew) stresses. Expression-based studies suggested their responsive role against heat and powdery mildew stress [87]. Moreover, another study [96] highlighted the crucial role of the *AtHB1* gene in iron homeostasis. Therefore, it could be of great importance to examine the role of other members under nutrient starvation and homeostasis. Heavy metals such as Cd are increasing in the soil due to the massive industrial waste and mineralization of rocks. The uptakes of these heavy metals by major food crops are harmful not only to plant but also to human health. The HD-ZIP III subfamily gene OsHB4, when silenced, significantly reduced the Cd accumulation in rice grain. Therefore, it can be used as a potential biomarker to curb the toxic effects of Cd on plants and humans. Altogether, the genetic manipulation of HD-ZIP genes could be a handful strategy to maximize the crop yield under the looming threat of climate change using state-of-the-art genome-editing tools like the CRISPR/Cas system. 

## Figures and Tables

**Table 1:** The HD-ZIP family genes and their potential role in providing resistance against abiotic stresses.

Stress Control	Plant Species	Gene	Functions	References
	Subfamily I			
Drought	*Arabidopsis thaliana*	*AtHB7*	Overexpression of *AtHB7* regualte the expression of drought stress-specific genes.	[126]
	*Arabidopsis thaliana*	*AtHB13*	The *AtHB13* work upstream of the *JUB1* gene to confer drought stress.	[127]
	*Arabidopsis thaliana*	*HaHB11*	The *HaHB11* transgenic plants closed their stomata faster and lost less water than controls.	[128]
	*Alfalfa*	*HaHB11*	Longer roots and rolled leaves in *HaHB11* transgenic *alfalfa* plant.	[128]
	*Arabidopsis thaliana*	*AtHB12*	Nullify the negative effects of ABA signaling genes (*PYL5* and *PYL8*).	[129]
	*Oryza sativa*	*OsHOX4*	The *OsHOX4* modulate GA signaling by interacting with DELLA-like genes and GA oxidase genes.	[60]
	*Oryza Sativa*	*OsHOX22*	Higher expression of *OsHOX22* gene under drought stress.	[16]
	*Nicotiana attenuate*	*NaHD20*	Augmented ABA accumualtion in leaf.	[62]
	*Triticum aestivum*	*TaHDZ5-6A*	*TaHDZ5-6A* transgenic plants displayed enhanced drought tolerance by lowering the water loss rates, higher survival rates, and higher proline contents.	[9]
Salinity	*Arabidopsis thaliana*	*HaHB11*	Higher expression of salt stress-related genes.	[128]
	*Alfalfa*	*HaHB11*	Strong root activities.	[128]
	*Oryza sativa*	*OSHOX22*	Regualted ABA signaling.	[66]
	*Physic nut*	*JcHDZ07*	Overexpression of *JcHDZ07*-induced sensitivity to salinity stress.	[70]
	*Zea mays*	*ZmHDZ10*	Lower relative electrolyte leakage (REL), lowee MDA and increased proline content in overxpressed *ZmHDZ10* transgenic plant.	[69]
Heat stress	*Soybean*	*HaHB4*	*HaHB4* transgenic plant possesses larger xylem area, and increased water use efficiency under high temperature stress.	[11]
	*Perennial ryegrass*	*LpHOX21*	Higher expression of *LpHOX21* gene was recorded in heat-tolerant cultivar.	[84]
Heavy metal (manganese)	*Citrus sinensis*	*TDF #170-1, 170-1k*	Induced expression of these genes were observed under heavy metal stress.	[80]
Cold stress	*Triticum aestivum*	*TaHDZipI-2*	Frost toelrance-related genes were upregualted in *TaHDZip1-2* overxpressed plants.	[74]
	*Triticum aestivum*	*TaHDZipI-5*	Induction in lipid biosynthesis genes induced cold tolerance.	[75]
	*Arabidopsis thaliana*	*AtHB13*	Higher antioxidant activities of *AtHB13* transgenic plants.	[59]
	*Arabidopsis thaliana*	*AtHB1/HaHB1*	Induction of pathogenesis-related and glucanase proteins.	[76]
Flooding stress	*Arabidopsis thaliana*	*HaHB11*	Modulation of genes genes involved in glycolisis and fermentative pathways.	[87]
Nutrient stress (iron deficiency)	*Arabidopsis thaliana*	*AtHB1*	Overexpression of *AtHB1* regualtes iron homeostasis.	[92]
	Subfamily II			
Drought	*Sesame*	*SiHDZ13, SiHDZ42*	Higher expression under drought stress.	[93]
	*Triticum aestivum*	*Tahdz4-A*	Upregualted mRNA level under drought stress.	[94]
	*Eucalyptus*	*EcHB1*	Increased the leaf photosynthesis.	[98]
	*Arabidopsis thaliana*	*HAT2, HAT22*	High response to hormonal treatment.	[95]
Salinity	*Camellia sinensis*	*CsHDZ15, CsHDZ16*	Augmented expression under salinity stress.	[105]
	*Solanum tuberosum*	*StHOX17, StHOX20, StHOX27*	Higher expression under salinity stress.	[106]
	*Capsicum annum*	*CaHB1*	Upregulation of multiple genes involved in plant osmotic stress resistance.	[10]
Light stress	*Arabidopsis thaliana*	*AtHB2/HAT4*	Stimualted expression of phytochrome genes in overexpressed *AtHAT4* gene transgenic plant.	[100,130]
	Subfamily III			
Drought	*Oryza sativa*	*OsHB4*	LeafrRolling and altering stem xylem development.	[107]
Salinity	*Triticum aestivum*	*Tahdz1, Tahdz23*	Induced mRNA level under salinity stress.	[94]
	*Medicago truncatula*	*MtHDZ5, MtHDZ13, MtHDZ22*	Higher expression under salinity stress.	[109]
Cadmium stress	*Oryza sativa*	*OsHB4*	Silencing of *OsHB4* gene reduced Cd accumulation in the leaves and grains.	[12]
	Subfamily IV			
Drought	*Oryza sativa*	*OsHDG11*	Transgenic rice plants had higher levels of abscisic acid, proline, soluble sugar, and reactive oxygen species-scavenging enzyme activities under stress.	[51]
	*Chinese kale*	*AtEDT1/HDG11*	Induced stomatal closure.	[115]
	*Gossypium herbaceum*	*HDG11*	Augmeneted proline content, soluble sugar content, and activities of reactive oxygen species-scavenging enzymes.	[117]
	*Nicotiana tobaccum*	*NtHD-ZIP IV 4, NtHD-ZIP IV 10*	Higher expression under drought stress.	[119]
	*Nicotiana tobaccum*	*NtHDG2*	Induced flavonoid biosynthesis.	[131]
	*Oryza sativa*	*OsTFIL*	Promotes lignin biosynthesis and stomatal closure.	[116]
Salinity	*Gossypium herbaceum*	*AtEDT1/HDG11*	Better proline content, soluble sugar content.	[117]
	*Arabidopsis thaliana*	*EDT1/HDG11*	Promotes lateral root formation in *Arabidopsis* mutant edt1 by upregulating jasmonate biosynthesis.	[123]
	*Nicotiana tobaccum*	*NtHDG2*	Higher antioxidant activities.	[131]
Osmotic	*Arabidopsis thaliana*	*GaHDG11*	Upregualted expression level was observed under osmotic stress.	[125]

**Table 2:** List of functionally characterized HD-ZIP family genes under biotic stress.

Subfamilies	Plant	Gene	Pathogen	Functions	Reference
Subfamily I	*Arabidopsis thaliana*	*AtHB13*	Powdery mildew (*Odium neolycopersici*), downy mildew (*Hyaloperonospora arabidopsidis*)	Overexpression of *AtHB13* stimualted the expression of various defense related genes.	[138]
	*Zea mays*	*HaHB4*	*Spodoptera littoralis*	Modulate signals from the jasmonic acid and ethylene pathways.	[139]
	*Gossypium hirsutum*	*GhHB12*	*Verticillium dahliae*	Increased susceptibility of the cotton plant via suppression of the jasmonic acid (JA)-response genes *GhJAZ2* and *GhPR3*.	[141]
Subfamily II	*Solanum tuberosum*	*StHOX28, StHOX30*	*Phytophthora infestans*	Induced expression pattern under *Phytophora infestans.*	[106]
	*Capsicum annuum*	*CaHB1*	*Phytophthora capsici*	Overexpression of *CaHB1* in tomato resulted in a thicker cell wall.	[10]
Subfamily III	*Solanum tuberosum*	*StHOX7, StHOX16, StHOX26, StHOX38*	*Phytophthora infestans*	Induced expression pattern under *Phytophora infestans.*	[106]
	*Arabidopsis thaliana*	*AtHB8*	*Meloidogyne incognita*	The promoters of procambial marker gene *ATHB8* were activated in *M. incognita*-induced galls.	[146]
	*Arabidopsis thaliana*	*PHB, PHV*	TYLCCNV	Suppress selective jasmonic acid responses.	[147]
Subfamily IV	*Zea mays*	*ZmOCL1*	*Pseudomonas syringae*	Overexpression of *ZmOCL1* induced antifungal activity of a lipid transfer proteins.	[42,159,160]
	*Solanum tuberosum*	*StHOX21, StHOX42*	*Phytophthora infestans*	Induced expression pattern under *Phytophora infestans.*	[106]

## Data Availability

Not applicable.
